# A Comprehensive Review of Deep Learning in Computer Vision for Monitoring Apple Tree Growth and Fruit Production

**DOI:** 10.3390/s25082433

**Published:** 2025-04-12

**Authors:** Meng Lv, Yi-Xiao Xu, Yu-Hang Miao, Wen-Hao Su

**Affiliations:** College of Engineering, China Agricultural University, 17 Qinghua East Road, Haidian, Beijing 100083, China; lv.meng@cau.edu.cn (M.L.); 2022307140224@cau.edu.cn (Y.-X.X.); 17319137597@163.com (Y.-H.M.)

**Keywords:** computer vision, target recognition, smart orchard, apple tree growth, fruit production

## Abstract

**Highlights:**

**Q: What are the main findings?**

- Reviewed the use of deep learning techniques for monitoring and predicting apple pests, diseases, organ growth, yield, and defects.
- Reviewed more than 100 literatures from the past 7 years.
- Summarized the current state of the relevant literature in each part and proposed solutions to the problems.

**Q: What is the implication of the main finding?**

- Provided a reference for future research and drove the development of smart orchards.

**Abstract:**

The high nutritional and medicinal value of apples has contributed to their widespread cultivation worldwide. Unfavorable factors in the healthy growth of trees and extensive orchard work are threatening the profitability of apples. This study reviewed deep learning combined with computer vision for monitoring apple tree growth and fruit production processes in the past seven years. Three types of deep learning models were used for real-time target recognition tasks: detection models including You Only Look Once (YOLO) and faster region-based convolutional network (Faster R-CNN); classification models including Alex network (AlexNet) and residual network (ResNet); segmentation models including segmentation network (SegNet), and mask regional convolutional neural network (Mask R-CNN). These models have been successfully applied to detect pests and diseases (located on leaves, fruits, and trunks), organ growth (including fruits, apple blossoms, and branches), yield, and post-harvest fruit defects. This study introduced deep learning and computer vision methods, outlined in the current research on these methods for apple tree growth and fruit production. The advantages and disadvantages of deep learning were discussed, and the difficulties faced and future trends were summarized. It is believed that this research is important for the construction of smart apple orchards.

## 1. Introduction

Apples have become one of the most popular fruits due to their richness in vitamins and minerals. It is known that the total annual production of apples has reached 87.2 million tons, making it the third most produced fruit in the world after bananas and watermelons [1]. Therefore, ensuring the healthy growth of apple fruit trees and the production of quality fruit is of great importance for the development of the world economy. However, most of the orchard operations are still done manually, which requires a lot of labor and learning costs and is extremely detrimental to the development of orchards. Traditional machine learning algorithms have been used to detect fruit tree growth and fruit yield, but these algorithms are difficult to meet real-time requirements in practical applications due to high computational complexity and slow processing speed. In large-scale orchard management, real-time detection is extremely important. By reducing computational latency and speeding up data processing, real-time detection enables rapid feedback and improved processing efficiency, thereby enhancing the scalability and utility of large-scale orchard production.

**Figure 1:**
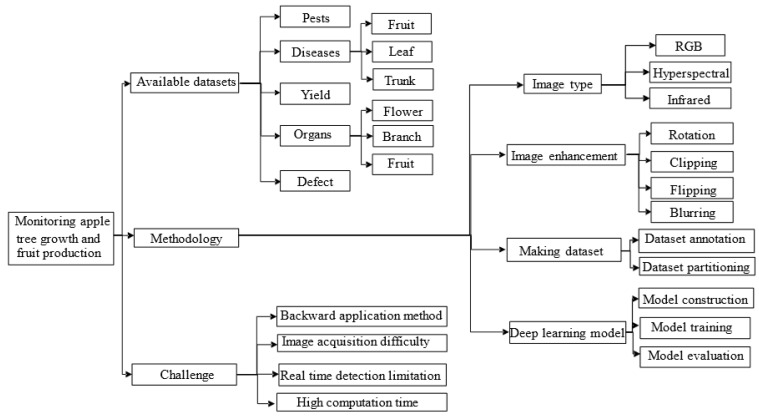
Summary of literature searches for monitoring apple tree growth and fruit yield based on deep learning and computer vision techniques.

The emergence of deep learning techniques has been successfully applied to solve real-time recognition and detection tasks in the field of computer vision, such as face recognition [2], traffic detection [3], motion recognition [4], and human pose estimation [5]. In recent years, it has shown great potential for monitoring fruit tree growth and automated orchard production. Previous detection methods mainly rely on traditional image processing algorithms or manually extracted features plus classifiers. The features extracted by traditional methods are mostly low-level, and it is difficult to extract high-level semantic features. In addition, the imaging methods are also carefully designed, resulting in low generalization ability of the algorithms, which are affected by lighting conditions, background changes, occlusion, and other factors in practical use, leading to poor recognition and poor robustness. However, deep learning models can autonomously learn features from large datasets; researchers can adjust the structure of the network model to improve the training process of the model according to their needs, thus improving the prediction results of the model, which promises to automate orchard production. For deep learning, more accurate images can help models extract features better, and using different imaging techniques can help to obtain more complex image features [6]. Common imaging techniques, such as red-green-blue (RGB) imaging, thermal infrared imaging, spectral imaging, and three-dimensional (3D) imaging, have been widely used in automated inspection. In Section 4.3, regarding the detection of apple branches, the 3D point cloud technique is applied to effectively extra the depth information of branches to solve the problem of branch occlusion. As early defects in apples are hidden in the peel tissue and are difficult to detect by the human eye or common RGB imaging techniques, Section 4.5 describes the use of imaging techniques, such as hyperspectral images, near-infrared images, and thermal imaging images combined with deep learning to detect early defects. The use of a deep learning model combined with different imaging techniques to monitor apple tree growth and fruit production is illustrated in Figure 1.

In recent years, deep learning has been widely used to study various aspects of apple orchard production operations and build reliable artificial intelligence (AI) systems. RGB, spectral, and other types of images collected from orchards were analyzed with the aim of automating orchard operations in real time. Bonkra et al. [7] explained in detail segmentation models and detection methods for apple leaves from an architectural perspective and summarized the research on deep learning techniques for apple leaf diseases. Villacrés et al. [8] reviewed research on deep learning techniques for yield prediction in apple orchards. In addition, studies on apple tree pruning [9], fruit detection, classification, and grading [10] have been reviewed accordingly. Teixeira et al. [11] reviewed the use of deep learning techniques for automated detection, localization, and classification of insects and summarized the existing challenges. However, the factors affecting apple fruit quality are multifaceted, and a single review is insufficient for the development of the apple industry. To date, there is no comprehensive review literature summarizing the use of deep learning techniques for monitoring the whole apple growth process and fruit quality. This study outlines the need to use deep learning combined with computer vision techniques to monitor apple tree growth as well as fruit production. The second section focuses on computer vision techniques. The third section introduces deep learning techniques. In the fourth section, the current state of research on apple pests, disease detection, organ growth detection, yield prediction, and post-harvest defect detection is described. The advantages and disadvantages of deep learning techniques are also discussed, along with challenges and future trends, which are accompanied by possible solutions to existing problems.

## 2. Computer Vision

The principle of computer vision technology is to replace the human perception organs with various imaging devices and the human brain with a computer system, which can simulate human observation and understanding of the world independently. Computer vision technology consists of two main components: hardware-based image acquisition and software-based image processing [12]. Image acquisition relies on hardware such as cameras, lighting systems, and sensors, where the quality of captured images directly influences downstream tasks [6]. Image processing includes various computational techniques, such as filtering, segmentation, and feature extraction, to enhance image clarity and extract meaningful information. Computer vision technology has become an essential tool in modern agriculture, enabling automated and precise visual analysis for improved crop management.

## 3. Deep Learning

Deep learning is a subfield of AI that has led to revolutionary advances in computer vision by automatically extracting features and performing pattern recognition from large-scale datasets. Deep learning relies on multilayer neural networks, particularly convolutional neural network (CNN) and transformer-based architectures, to be able to learn hierarchical representations from image data. These models typically include four key phases: data acquisition, data preprocessing, model training, and performance evaluation [13]. As technology continues to evolve, deep learning methods have demonstrated significant benefits in several domains, especially in complex and dynamic environments, with higher accuracy and adaptability. Through automated feature learning, deep learning models can efficiently process large-scale data, thereby improving efficiency and accuracy in computer vision tasks. With excellent robustness and flexibility, deep learning is particularly suitable for application in real-world complex scenarios, which can optimize the decision-making process and drive innovation in various fields.

## 4. Monitoring of Apple Tree Growth and Fruit Production

Compared to traditional machine learning algorithms or relying on manual, deep learning combined with computer vision is expected to achieve accurate detection and fast operation. This section provides an overview of the applications of deep learning combined with computer vision techniques in five areas—pest detection, disease detection, organ growth detection, yield prediction, and post-harvest defect detection—and summarizes the current state of research in each area.

### 4.1. Pest

Pests can affect the yield and quality of plants, and even the ecology, so pest monitoring is an important part of plant production. In the past, manual detection was the most used means of pest monitoring. However, manual detection requires high labor and learning costs, making pest monitoring difficult and time-consuming. In recent years, deep learning combined with computer vision has achieved excellent results in various tasks, especially in image classification tasks, which have far surpassed humans [14]. Deep learning combined with computer vision has been applied to various fields for their powerful feature extraction capabilities and has also achieved remarkable results in the field of pest detection, providing a new development direction for real-time pest detection research.

**Figure 2:**
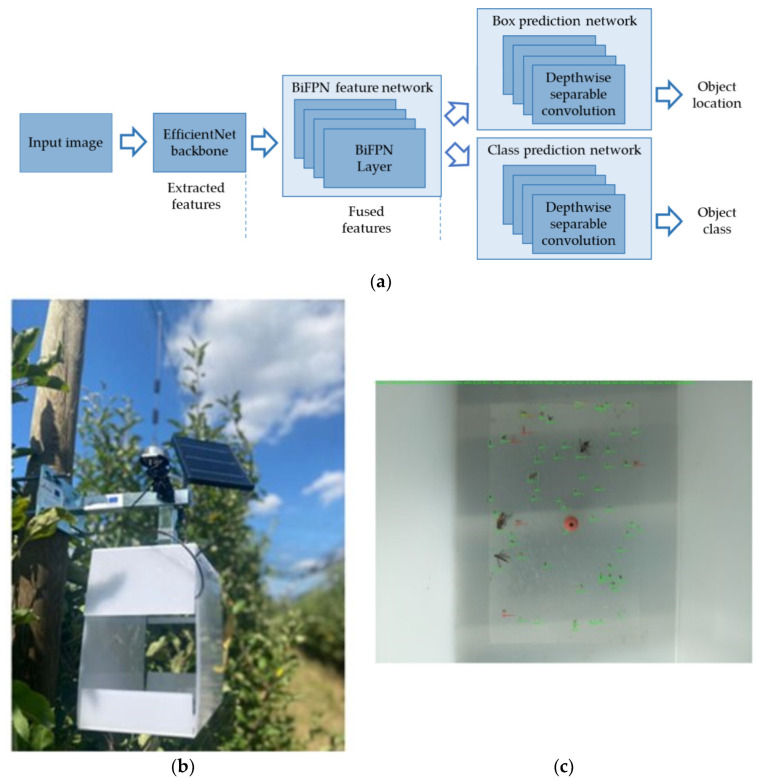
The PMD for identification of pests. (**a**) The structure of the EfficientDet neural network. (**b**) The PMD. (**c**) Automatic counting results for PMD models (red border box—detecting classe malifoliella, green border box—detecting classes other insects) [27].

Common pests of apples include apple woodlouse, apple weevil, apple moth, apple borer, and apple aphid. Several scholars have studied the corresponding classification models. For example, Boniecki et al. [15,16] agreed that multilayer perceptron (MLP) has better capabilities in pest identification and classification tasks, whereas Zaborowicz et al. [17] compared MLP with radial basis function (RBF) and deep neural networks (DNN) classification models and concluded that the pest identification problem is potentially linear in nature. In order to solve the problem of background clutter and uneven illumination in real environments, Wen et al. [18] introduced the pose estimation-dependent automatic recognition algorithm to deep networks and compared it with several classical supervised learning methods, such as support vector machines (SVM), logistic regression classifier (LRC), BayesNet, RBF, and random forest (RF), which eventually yielded good results. Janarthan et al. [19] experimented by introducing a dual-attention mechanism into the MobileNetv2 model by constructing three different-sized datasets, which eventually achieved an average accuracy of over 90.00% through fivefold cross-validation. Aghajanpoor et al. [20] applied a transfer learning approach to the identification of a pest such as powdery mildew by applying the method to three different convolutional neural network architectures, including visual geometry group (VGG16), AlexNet, and GoogLeNet, which showed that AlexNet had a higher accuracy rate of 99.53%. Dong et al. [21] built the PestLite model based on You Only Look Once, version 5 (YOLOV5). The PestLite model used the multi-level spatial pyramid pooling (MTSPPF) to replace the original spatial pyramid pooling fusion (SPPF) structure of YOLOV5 and introduced the efficient channel attention (ECA) model to enhance the understanding of the overall context and adopts content-aware reassembly of features (CARAFE) to replace the traditional upsampling structure. The mean average precision (MAP) of the PestLite model is improved by 2.80% compared with that of the YOLOV5 model, and the number of participants is reduced from 7.03 million to 6.09 million. In addition, it is of great practical importance to carry out research on automatic capture devices. In order to find fast and accurate pest detection techniques, Albanese et al. [22] and Segalla et al. [23] compared the performance of LeNet and VGG16 and found that LeNet had better recognition accuracy, making it more suitable for application in embedded systems. Brunelli et al. [24] designed an automatic detection device; the results showed that the VGG16 model used could achieve 92.60% accuracy, with the lowest average energy consumption due to its low system duty cycle and low hardware cost. Abbaspour-Gilandeh et al. [25] introduced a sparse coding algorithm and combined it with an artificial neural network (ANN); the algorithm achieved 90.00% pest detection accuracy. Suárez et al. [26] proposed the use of an image processing approach for feature extraction and a CNN classifier to classify pests in actual fields. Pest management by judging whether the number of detected insects reached a threshold, and the results showed that the overall accuracy of the classifier was as high as 94.80%. Čirjak et al. [27] used the EfficientDet model to identify malifoliella, healthy leaves, and dirt with up to 98.00% accuracy and then integrated the model into a pest monitoring device (PMD) for real-time identification and capture of pests, as shown in Figure 2. To address the pest adhesion problem that exists in orchard pest detection, Wang et al. [28] proposed a segmentation followed by identification method. Based on the segmentation method of density curvature weighted Gaussian mixture model (GMM-DC) and later improved Mask R-CNN model as the recognition model, this method has a substantial improvement in the recognition of adherent pests, and the average accuracy can reach 96.75%.

A summary of deep learning-based pest detection is shown in Table 1. In terms of pest detection and classification, MobileNetv2 can achieve good results with a smaller number of parameters compared to VGG16 and LeNet. Many researchers have focused on the accuracy of the model while reducing the weight size and hardware cost of the model by using lightweighting algorithms, and the current mainstream lightweighting algorithms, such as knowledge distillation and pruning, are expected to be applied. What should not be overlooked is that there are still some problems in this area. For example, the algorithms currently used in this field are relatively old, and some new methods, such as contrast learning and vision transformer (VIT), are less applied, which may provide new solutions for pest detection. In addition, the difficulty in acquiring pest images due to the seasonal and random nature of pests, which is one of the main reasons why most of the current research on pest detection equipment is stuck in the laboratory stage, can be solved by introducing advanced techniques, such as semi-supervised learning, active learning, and data enhancement.

### 4.2. Diseases

Diseases are likewise one of the main causes of reduced apple yields. Timely detection and accurate identification of disease types so that effective control measures can be taken and precise fertilizer applied is a development objective for smart orchards. Therefore, to ensure the healthy growth of apple trees, the timely detection of lesions on their fruit, trunk, and leaves is essential. In fact, when these diseases occur, they are often vastly different from healthy surfaces. Image acquisition by optical sensors and then image processing using computer vision techniques can detect apple diseases in complex backgrounds and lighting environments, but they cannot be accurately classified [29]. Advanced deep learning techniques combined with computer vision have successfully solved this problem, making automatic detection and accurate classification of diseases possible.

Based on the detection of apple fruit diseases, many scholars have done related studies. Rahul Sharma et al. [30] and Tian et al. [31] both used the CycleGAN method to extend the dataset and then used the improved YOLOV3 deep learning model to detect anthracnose on the apple surface with an intersection over union (IOU) of more than 95.00%. Gu et al. [32] introduced the k-nearest neighbors (KNN) algorithm into CNN; the KNN algorithm can extract more powerful and in-depth features to classify the three diseases, and the accuracy can reach 99.78%. Ayaz et al. [33] proposed DCGAN-DCNN by fusing deep convolutional generative adversarial network (DCGAN) and deep convolutional neural network (DCNN) with different architectures. The DCGAN model generates a new original image used to overcome the limitations of apple disease images. The DCNN model consists of five convolutional, two dense, and one decision vector layer, which is for classification of apple diseases. The DCGAN-DCNN model achieved 99.99% accuracy in classifying different apple diseases compared to models such as ResNet, SqeezeNet, and MiniVGGNet. Zhang et al. [34] proposed a dual-channel convolutional neural network (DMCNN), which transformed the input image into two color spaces, RGB and hue-saturation-value (HSV), and used the dual channels to extract color and texture features, respectively, through training on 5010 images; the classification accuracy can reach 99.50%.

**Figure 3:**
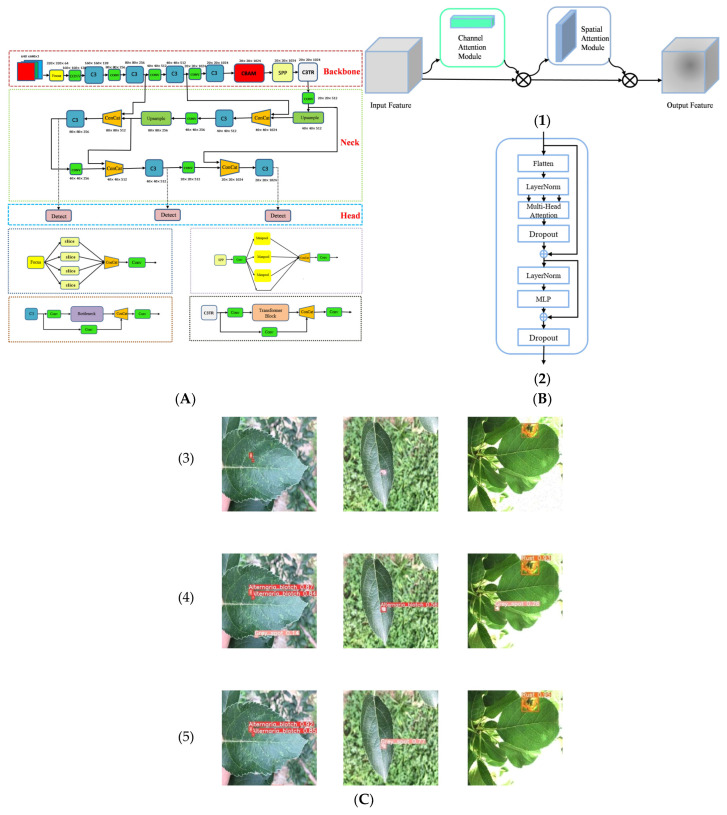
Accurate localization and identification of apple leaf diseases using YOLOV5-CBAM-C3TR. (**A**) The overall architecture of YOLOV5-CBAM-C3TR. (**B**) The detailed structure of the added optimization module: (**1**) CBAM, (**2**) Transformer. (**C**) Comparison of recognition results of different models in real scenes: (**3**) original image, (**4**) recognition results using YOLOV5, and (**5**) recognition results using YOLOV5-CBAM-C3TR [39].

Apple leaf diseases spread rapidly and cause huge yield losses in a short time; many scholars have studied leaf diseases, especially the classification of diseases. Yu et al. [35] proposed a region of interest (ROI) based on DCNN method, and the ROI subnetwork could improve the recognition accuracy of leaf diseases by its dataset containing 404 images of Marssonia blotch disease and Alternaria disease, which enabled the recognition accuracy to reach 84.30%. Singh et al. [36] compared four classical machine learning algorithms, including SVM, KNN, random forests, and logistic regression models, as well as three improved CNN networks proposed by themselves to classify two diseases in 20,000 apple leaf disease samples. They combined various performances in practical development system applications, and finally, the CNN-C achieved 99.20% accuracy and 99.70% sensitivity. Based on the Densenet-121 deep convolutional network, Zhong et al. [37] proposed three methods, including regression, multi-label classification, and focal loss function, to identify a total of 2462 images of six diseases, with accuracies of 93.51%, 93.31%, and 93.71%. This approach improves the recognition accuracy of deep learning networks in unbalanced datasets compared to combining deep convolutional networks with cross-entropy loss functions. Liu et al. [38] proposed an improved lightweight real-time leaf disease detection model based on YOLOX-Nano named YOLOX-ASSANano; they collected 6268 images containing three diseases named MSALDD by themselves and experimented with MAP of 91.08%. Lv et al. [39], introducing the convolutional block attention module (CBAM) and convolution 3 transformer (C3TR) into YOLOV5, proposed YOLOV5-CBAM-C3TR and found that the fusion of the two modules led to a substantial improvement in the recognition of three apple leaf diseases. The architecture of the model and the actual recognition effect are shown in Figure 3. In this study, it especially found that YOLOV5-CBAM-C3TR showed better recognition of similar diseases. Fu et al. [40] proposed an improved VIT model, which effectively selects the more obvious regions by chunking, while the transformer mechanism can better focus on less obvious features. The experimental results on a dataset containing seven samples resulted in a MAP of 84.00%, but the model is susceptible to sample imbalance in the dataset, resulting in poor identification of species with small samples. In addition, some scholars have attempted practical deployments to solve the real-time disease recognition problem. Özden et al. [41] proposed a new migration learning approach by applying background removal and some enhancement techniques to approximate two datasets, namely the Phytopathology dataset and the PlantVillage dataset. They pretrained the MobileNetV2 on this combined dataset, and five optimizers—Adam, Adagrad, Adadelta, PowerSign, and RMSProp—were compared. Finally, the MobileNetV2 converted to a TFLite model and deployed in a mobile application, achieving a high accuracy rate. However, the camera could only be kept in a certain position when performing recognition to avoid interference from the surrounding environment. Bansal et al. [42] proposed an ensemble of pretrained DenseNet121, EfficientNetB7, and EfficientNet, which was experimented on a dataset of 3642 images containing apple scab, apple rust, and multiple diseases. This ensemble model achieved an accuracy of 96.25% and finally deployed on a simple web application. Upadhyay et al. [43] extracted apple leaf diseases by preprocessing the original images with image segmentation using the GrabCut method and recognized the diseases by the improved ResNeXt. By training on 9395 datasets with different kinds of apple diseases, the improved ResNeXt model finally achieves 98.94% accuracy for the four kinds of apple leaf diseases. Kumar et al. [44] proposed an optimized deep learning model by improving the network structure of the ResNet model and integrated it into an application for real-time detection of apple leaf diseases. The model showed a good detection effect on the dataset, including three kinds of apple leaf diseases. Wang et al. [45] optimized the ELM-YOLOV8n based on the advanced YOLOV8n by integrating the Fasternet Block and exponential moving average (EMA) attention mechanism and finally proposed the ELM-YOLOV8n. Compared with the original model, the MAP of ELM-YOLOV8n in detecting leaf diseases was increased to 96.70%, and the number of parameters and computational load were reduced by 44.80% and 39.50%, respectively. This significantly enhances the ability of small target disease detection in complex backgrounds and the feasibility of mobile deployment. To achieve accurate detection of apple leaf diseases, Fan et al. [46] proposed a semi-supervised method as pyramid scene parsing network self-attention (PSPNet-SA), which mean intersection over union (MIOU) scores of 0.975, 0.974, and 0.965 using only 1/2, 1/4, and 1/8 of the annotated data, respectively. The improved PSPNet-SA reduces the dependence on large annotated datasets and computational complexity while maintaining high recognition accuracy for apple leaf diseases. Further, a transformer-based fine-grained multi-label classification framework was developed with F1 scores of 0.855, 0.903, 0.919, 0.921, and 0.895 for Alternaria blotch, brown spot, grey spot, mosaic, and rust, and this model performed well in evaluating the different disease classifications.

Some scholars have studied the detection of apple tree trunk diseases. Compared with AlexNet, VGGNet, GoogLeNet, ResNet, and squeeze-and-excitation networks (SENet), the proposed improved loss function model [47] based on VGG19 can obtain an accuracy of 94.50%, but the accuracy of the classifier is influenced by the quality of the input image. Guan et al. [48] combined descriptive text features with image features and proposed a CNNDNN-BiLSTM model, a pest diagnosis model that combines representation learning with a two-channel neural network, which was used to test for three diseases, ultimately achieving an 88.00% accuracy rate.

To sum up, as shown in Table 2, deep learning techniques have been used for disease detection and classification of apple fruits, leaves, and trunks. Although the diseases are distributed in different parts of the fruit tree, the principles of disease identification and detection are the same. The advanced DCNN, VIT model, and YOLO series of models perform far better than image processing techniques in disease detection tasks, where the advanced VIT model and YOLO series of models are more effective in detecting similar diseases and can be used in complex environments. In addition, many researchers are investigating further deployment of models to mobile devices for real-time disease detection tasks [49,50]. Currently, there is an increasing number of studies on apple leaf diseases mainly because apple leaves are the largest area covered in the growth of fruit trees and play a key role in their healthy growth. Studies on diseases of fruit and trunk parts are still relatively scarce, mainly due to the difficulty in obtaining relevant images and the lack of publicly available datasets; studies on fruit and trunk disease classification are only in the laboratory stage. In the future, more comprehensive datasets and improved detection models are needed to quickly and accurately detect and classify common diseases and to mount spraying devices for accurate spraying of orchard diseases.

### 4.3. Organ

To safeguard the economic efficiency of growers and to help them optimize their orchard management, it is important to automatically monitor the growth of the various organs of the apple tree and to carry out timely horticultural work. Monitoring the growth of apple blossom in the early stages allows the density of fruit growth to be determined indirectly so that appropriate thinning operations can be carried out. Monitoring the growth of branches allows better pruning and tying operations to be carried out, while the detection and three-dimensional modeling of branches is a key step in the robotic harvesting of fruit for obstacle avoidance operations. Monitoring the growth of apple fruit, such as size, ripeness, color, and other growth characteristics, can be determined so that timely action can be taken to ensure orchard production. Deep learning combined with computer vision can obtain the accurate field information needed, promising to automate orchard management and thus maximize the benefits for orchard growers.

**Figure 4:**
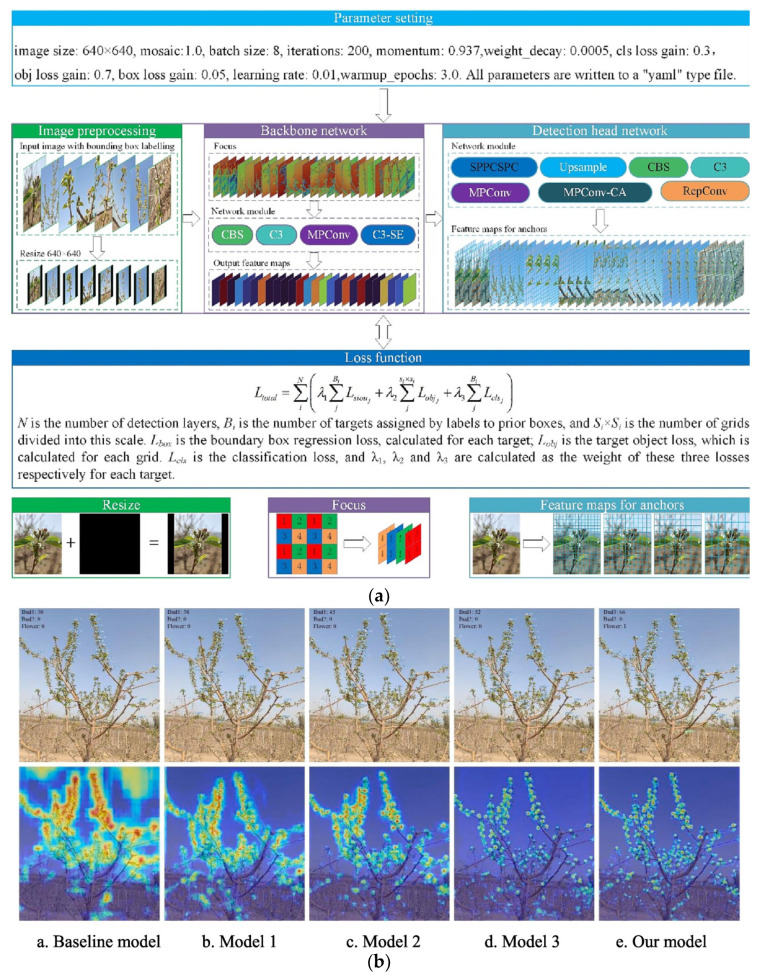
The improved YOLOV7 model for identifying apple blossoms. (**a**) Parameter configuration and overall structure diagram of the improved YOLOV7 model. (**b**) Comparison of the accuracy of different models for detecting apple blossoms using thermodynamic force maps [57].

Unlike previous methods based on color thresholds to detect apple blossoms, which are highly susceptible to factors such as lighting conditions and shadows [51], many scholars have conducted research based on deep learning techniques. Dias et al. [52] proposed a CNN-based apple blossom detection algorithm and conducted experiments on AppleA dataset (consisting of 147 images containing apple blossoms), which achieved an accuracy of 92.70%. By conducting experiments on three other datasets with different lighting and flower types, it found this algorithm accurately identifies apple blossoms with a best recall and accuracy of around 80.00%. Dias et al. [53] used the region growth segmentation (RGR) algorithm to refine the output of the DeepLab model using a deeper CNN network by segmenting an AppleA dataset containing 124 images for segmentation, achieving an accuracy of 79.40%. Bhattarai et al. [54] applied the Mask-RCNN algorithm to the detection of apple blossoms. Using different image enhancement techniques to improve the accuracy of the model, ultimately achieving an average accuracy of 86.00% on a dataset containing 9691 apple blossoms. To address the instability and overfitting of convolutional neural networks in the case of insufficient samples, Zhang et al. [55] used generative modules and image preprocessing methods for data enhancement and then reduced the model parameters by pruning. Comparing the YOLO series, SSD series, and EfficientDet series models, they proposed the GM- EfficientDet-D5 model, which achieved 90.01% apple blossom detection accuracy with an inference speed of 29 FPS for apple blossom detection. Shang et al. [56] introduced ShuffleNetv2 and Ghost lightweight modules into the YOLOV5s model to propose the YOLOV5s-ShuffleNetv2-Ghost model, which has a MAP of up to 91.80%, and the detection speed of the model has been greatly improved. In addition, the number of parameters of the model has achieved a significant reduction in the real-time accurate monitoring of apple blossom. Based on YOLOV5, Chen et al. [57] used the YOLOV7 as the base model by adding the SENet and coordinate attention (CA) modules to the backbone network, using the SIoU bounding box regression loss function and replacing the 80×80 detection header. The improved model has a MAP of 80.10% and a recognition speed of 42.58, resulting in optimal performance. The structure of the model and the detection results are shown in Figure 4, demonstrating the use of heat maps to compare the effectiveness of YOLOV7, three other improved YOLOV7 models and the proposed improved YOLOV7 model in accurately identifying apple blossoms. In addition, a recognition system for apple blossoms was developed for visual statistical analysis of the results.

Deep learning techniques have been used to detect branches of apple trees to automate branch pruning, bundling, and field picking for obstacle avoidance operations. The trunk and branches of apple trees are often difficult to distinguish due to their similar color. Majeed et al. [58] used Kinect V2 sensor (Microsoft, Redmond, WA, USA) to acquire the RGB and point cloud data of the tree, removed the background interference with the help of depth information, and then used SegNet model for semantic segmentation. The experiment was performed on a dataset containing 300 images containing branches, and an accuracy of 92.00% was obtained. Majeed et al. [59] applied the same approach on a dataset with 509 images with an average accuracy of 94.00%. Chen et al. [60] compared the original and improved models, including U-Net, DeepLabv3, and Pix2Pix, focusing on the segmentation of occluded branches by performing experiments on a dataset of 521 images containing depth images, and finally found that the DeepLabv3 model achieved higher branch detection accuracy. Deeplab v3+, ResNet-18, VGG-16, and VGG-19 CNN networks were experimented on 674 images (containing branches, trunks, fruits, and leaves); ResNet-18 showed better results with an average MAP of 97.00% [61]. To ensure the robustness of the model in real-world environments, Su et al. [62] collected a total of 1800 images in all seasons of the year and proposed an improved YOLOV5s target detection model with an average accuracy of 95.61%, 98.37%, 96.53%, and 89.61% in spring, summer, autumn, and winter.

Deep learning networks are also heavily used in the study of apple fruit detection. Risdin et al. [63] chose the CNN model to detect fruits and experimented on a dataset containing a total of 2403 images of four fruits, namely grapes, apples, lychees, and lemons, with an accuracy of 99.89%. Tian et al. [64] proposed an improved YOLOV3 model by introducing a feature layer with lower resolution in the DenseNet optimization model to improve the performance of the model. Fukuda et al. [65] proposed a central roundish object painter (CROP) algorithm based on U-Net network, which uses a noncontact method to automatically measure the size of fruits based on the provided RGB images. Conducting experiments on 172 fruit samples, including apples and pears, the IOU reached 97.50%. Wang et al. [66] developed a system for remote monitoring of apple fruit growth and proposed a fused convolutional feature network based on ResNet-50. The F1 score of this edge detection network was 53.10%, but the edge information of the target apple could be extracted effectively, and the system also showed good results when it was tested in actual application scenarios. To enhance the detection of apple fruit, both Sekharamantry et al. [67] and Zhao et al. [68] structurally optimized YOLOV5 model, and both showed improved results compared to the original model, which also proved to be more robust under the influence of light, noise, and other factors.

In conclusion, as shown in Table 3, ResNet, U-Net, and SegNet have been widely used in apple organ detection research, solving the problems of changing light conditions and environmental interference that cannot be handled by image processing algorithms. Introducing generative adversarial network (GAN) models into apple blossom detection can solve the problem of apple blossom detection in dense orchards by improving image quality. Combined with image depth information, it can distinguish branches and trunks in the orchard in addition to monitoring growth characteristics such as fruit size and maturity. 

### 4.4. Yield

Accurate apple yield forecasting is vital for both apple growers and sellers; growers can better control the fruit production and harvesting process; sellers can make better decisions by anticipating packaging and storage costs in advance. In the past, growers relied on manual data collection by randomly selecting a few trees as a sample and extrapolating the number of apple trees in the whole orchard. However, this method was inaccurate and was rather time-consuming [69,70]. Later, automated yield estimation using image processing algorithms were also used [70], but resulted in inaccurate predictions due to factors such as variation in natural lighting and fruit obscured by other objects such as leaves and branches. To achieve the accurately predicted yield, deep learning combined with computer vision was used to address this problem.

**Figure 5:**
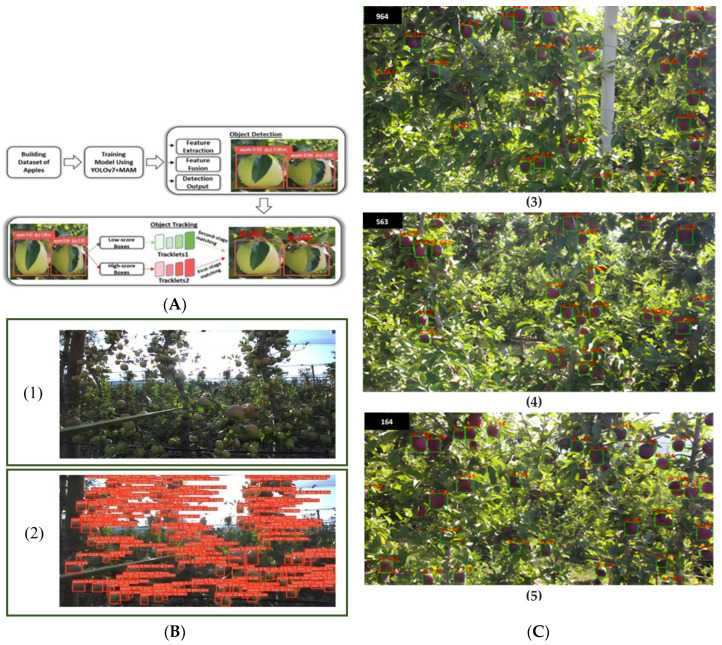
Counting apple production in the orchard using YOLOV7 + MAM. (**A**) The specific architecture of the YOLOV7 + MAM model. (**B**) Performance demonstration of YOLOV7 + MAM model for apple fruit recognition: (**1**) the original image in orchard, (**2**) the image of YOLOV7 + MAM model for identification apples in orchard. (**C**) The apple tracking results of the model for different video frames: (**3**) apple tracking results for Video ID1 using YOLOV7 + MAM + ByteTrack, (**4**) apple tracking results for Video ID2 using YOLOV7 + MAM + ByteTrack, (**5**) apple tracking results for Video ID3 using YOLOV7 + MAM + ByteTrack [75].

Many scholars have used deep learning techniques to predict apple orchard yields. Bargoti et al. [69] used multi-scale multilayered perceptron (MS-MLP) and CNN for image segmentation and then introduced watershed segmentation (WS) technique and circular hough transform (CHT) for pixel-level image processing for detection and counting of apples. The experiments showed that the F1 score of the algorithm was up to 86.10%. Zhang et al. [71] chose ResNet-10 as the backbone network, introducing path aggregation network (PANet) to deal with multi-scale features and using EfficientNet-64 as the classification network. The apple fruit yield can be detected while also estimating its maturity, with the lowest error rate compared to other methods based on deep learning techniques. Häni et al. [72] proposed a modular end-to-end orchard yield prediction system, which combined classical segmentation methods with a technology-based CNN approach to count orchard apples. Comparing the number of apples on multiple fruit trees with the total number of apples detected in experiments on three collected datasets with up to 97.83% accuracy. To better predict orchard fruit yield, Rao et al. [73] classified apple quality and proposed a CNN-SVM model through a mixture of traditional machine learning and deep learning. The CNN-SVM model can predict four types of apples representing high and low product varieties, and the accuracy of the model can reach 99.70%, which is a significant improvement compared with the method using CNN alone. However, single-target detection cannot reliably perform the task of fruit counting in an orchard. The reason is that if the same fruit appears in more than one image, the model repeats the counting, resulting in false positives. To address the challenges of occlusion of fruit counts and variability among the same fruits, Hu et al. [74] combined the self-attention mechanism of VIT with YOLOV7 and proposed YOLOV7-CA; the model achieved 91.30% of MAP and 0.85 of F1 scores, which significantly improved the accuracy of fruit yield prediction. Subsequently, to better improve the apple counting results, Hu et al. [74] used a multi-target tracking method based on Kalman filtering and motion trajectory prediction, which can accurately capture the number of apples between frames, while the detection speed of the model and the model size need to be further improved. Sekharamantry et al. [75] proposed to add the multi-attention mechanism (MAM) to YOLOV7 model to detect apples in the orchard and integrated ByteTrack to track multiple apples for counting task, which achieves high accuracy in apple detection and counting. This model architecture, performance comparison, and presentation of results are shown in Figure 5. Tan et al. [76] proposed the AppleYOLO model for accurate apple yield estimation. The AppleYOLO model integrates YOLOV8 with the Deep OC-SORT method, utilizing Fasternet as the backbone and incorporating focal modulation. In the feature fusion stage, it employs dynamic convolution KernelWarehouse and deploys the Deep OC-SORT method. On a custom dataset, the AppleYOLO model achieved MAP of 98.50%, significantly outperforming other methods. Bargoti and Underwood et al. [77] applied the Faster R-CNN model for fruit detection on a dataset of 2268 fruit images consisting of apples, mangoes, and almonds, mapped the detection results to yields, and performed object association between adjacent frames and proposed Tiled Faster R-CNN, which showed poor results in fruit-dense orchards. 

In short, as shown in Table 4, deep learning techniques have been widely used in fruit detection and yield prediction in orchards. However, there are still many problems with fruit counting and yield prediction. Problems such as fruit overlapping and double counting can lead to inaccurate counting results and, hence, incorrect yield prediction, which is expected to be solved by the combination of optimization of detection models and multi-objective tracking methods. In addition, it is impossible to make large-scale fruit yield predictions, which is mainly limited by the fact that researchers need to make manual counts and compare them with the detections of the model to assess the effectiveness of the model’s detection. Dynamic yield prediction is also a direction for future development; combining internet of things (IOT) counting with real-time detection to build a dynamic prediction model to predict fruit yield by collecting orchard fruit growth data will be more effective in predicting yield.

### 4.5. Defect

The quality of apples can be affected by external human or machine forces during planting, harvesting, and transport, which can lead to defects. When apples enter the market, they are sorted so that customers can select the apples of their choice. In the past, most sorting of apples was done by hand, which has been replaced by machine learning algorithms in recent years [78,79]. However, with the development of advanced technology, computer vision combined with deep learning techniques can automatically extract advanced features from the original image for accurate classification.

**Figure 6:**
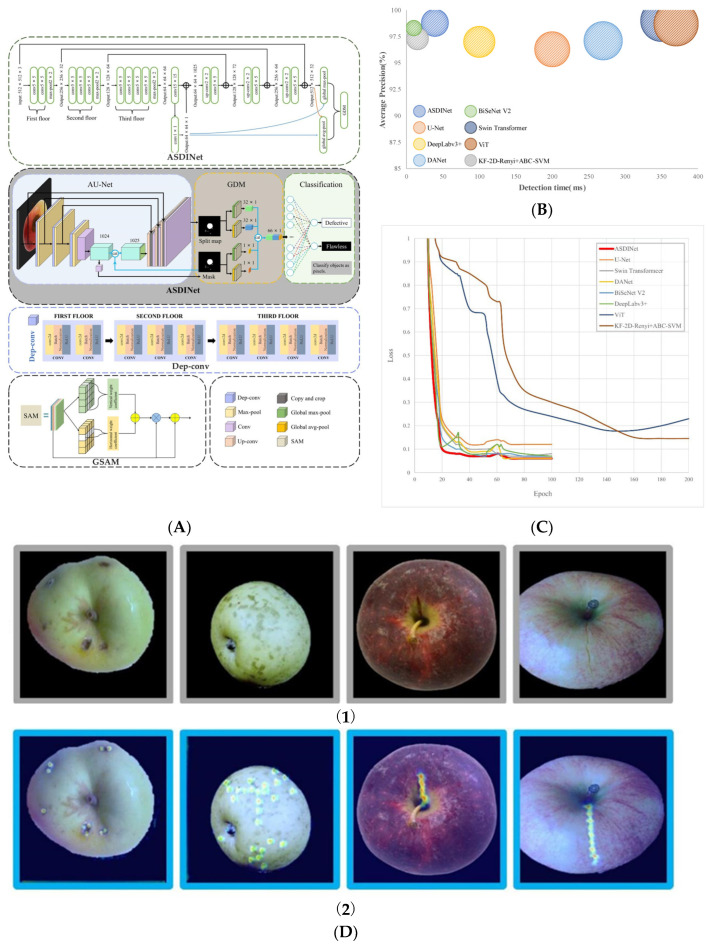
(**A**) Flowchart of apple fruit defect detection using ASDINet. (**B**) The comparison of AP metrics versus time with other SOTA models. (**C**) The comparison of loss metrics with other SOTA models. (**D**) Class activation map: (**1**) original image, (**2**) recognition results of apple fruit defects by U-Net [85].

Defects in apples, which occur due to bumps or scrapes, greatly affect the appearance of the apples and their sale. Many scholars have carried out studies accordingly. Lu et al. [80] obtained direct current (DC) and alternating current (AC) images on two varieties of apples using a multispectral structured-illumination reflectance imaging (SIRI) system. Comparing CNN, RF, and SVM, they concluded that CNN achieved an overall accuracy of 98.00%. Siddiqi et al. [81] compared two models, including SSD and YOLOV2, to defect detection by training and testing on a dataset consisting of 244 RGB images. They found the SSD model performed better. Li et al. [82] designed a CNN model to experiment on an apple dataset with 36,000 apple images with 99.00% accuracy. Stasenko et al. [83] introduced U-Net model and Deeplab model into CNN and training on 651 images containing apple fruits. The result showed that both models could achieve more than 99.00% of the MIOU. Yin et al. [84] proposed an OB-Net model based on Reset model by introducing an attention mechanism. The recognition accuracy of OB-Net model for overall classification could reach 95.64%. To solve the problem of low accuracy of apple fruit defect detection under small sample conditions, Hu et al. [85] designed an apple surface defect detection network (ASDINet) based on U-Net; the specific structure is shown in Figure 6. The ASDINet used the AU-Net to segment the image and gradient descent method (GDM) module to predict the defect image quickly with mask input. Comparing with other state-of-the-art (SOTA) models, the ASDINet model achieves an AP value of 98.80% and an F1 score of 97.75% on a dataset containing 500 samples, which is the best performance in terms of detection speed and accuracy. Ünal et al. [86] acquired RGB and near-infrared (NIR) images separately for 500 images and then trained them using three models, including AlexNet, InceptionV3, and VGG16. The result found that NIR images are very effective in detecting early apple bruises, thus concluding that NIR images are more suitable for industrial applications. Gao et al. [87] proposed a quality rooting system based on a deep learning algorithmic framework and compared it with traditional deep learning models, such as U-Net, SegNet, PSPNet, UNet++, DeepLabv3+, and high-resolution network (HRNet), which had an accuracy of up to 93.00%, but the generalisation ability needs to be further enhanced. Yang et al. [88] introduced EfficientNet as the backbone network based on YOLOV8, used weighted intersection over union (WIOU) loss function to calculate the rectangular box, and added ShuffleAttention to improve the feature extraction ability, and the improved model can effectively improve the detection ability of apple defects. Shan et al. [89] enhanced the YOLOV8n model by incorporating the space-to-depth convolution (SPD-Conv) module and the multi-scale empty attention (MSDA) module. Additionally, the original feature fusion method was replaced by the context-guided feature pyramid network (CGFPN), leading to the development of the SMC-YOLOV8n model. In complex environments, the SMC-YOLOV8n model improved the apple defect detection accuracy by 2.70%. Zhang et al. [90] combined SIRI with deep learning to detect defects in acquired DC, AC, and ratio transformation (RT) images using YOLOV8n. The YOLOV8n model was trained on a dataset containing 8000 images of different formats and finally achieved a detection accuracy of 99.12%, which provides a technological breakthrough for simultaneous recognition of multiple defects.

To summarize, different imaging techniques combined with deep learning for detecting apple defects are shown in Table 5. Early defect detection is crucial for fruit growth, and other imaging techniques, such as hyperspectral images, thermal images [91], and X-ray imaging [92], can also be used for internal defect detection. However, the detection of early defects is still inaccurate, and in the future, machine vision, spectral imaging, and ultrasonic detection can be integrated and analyzed using multimodal data to improve the accuracy of detection.

## 5. Discussion

Recently, deep learning has been successfully solving a variety of computer vision tasks and has also demonstrated excellent capabilities in monitoring various aspects of apple tree growth. Deep learning techniques can accomplish problems that traditional machine learning algorithms cannot, especially in image processing, and one of the main techniques used to achieve this is CNN [94]. For example, in the pest detection section, CNN has better results after extensive learning compared to traditional ANN networks, which have proven to be more suitable for the development of embedded systems. CNNs combined with computer vision techniques can perform tasks such as target detection, segmentation, and classification. It can accurately detect diseases (located in leaves, trunk, and fruit) and can also classify diseases through semantic segmentation with an accuracy rate of over 90.00%. In addition, it can accurately count to predict orchard yields. Notably, CNNs can also be used for early defect detection by combining different imaging techniques. Overall, there is great potential for real-time fruit tree growth monitoring using CNN, one of the most widely used deep learning tools.

The most notable feature of deep learning is automatic feature extraction by constructing multilayer networks [95]. It has been shown that as the number of network layers increases, the prediction accuracy of the model becomes better. For example, ResNet-50 [96] and ResNet-101 [97] were chosen for the detection of apple leaf diseases, and the result showed that the accuracy of ResNet-101 improved by 5.00% over ResNet-50. However, it should not be overlooked that network models with high-level numbers often lead to complex parameters. For example, in the model optimization session, the data and weights needed to be adjusted are also relatively large, so the balance between model accuracy and parameters is very important. In addition, models require data to learn and are prone to overfitting if the amount of data is too small. The models only show high accuracy predictions on well-trained datasets, and once experimented on less similar datasets, poor results are obtained. This highlights the importance of dataset diversity in improving the generalization ability of deep learning models. In agricultural applications, the characteristics of the target object may vary significantly depending on factors such as variety, geography, and growing environment. It is often difficult for single variety datasets to cover these diversities, which may lead to overfitting to specific scenarios and exhibiting low robustness and adaptability in real-world applications. However, large amounts of data need to be accurately annotated by experts with some expertise or annotated volunteers, requiring significant time and labor costs. A similar issue is observed in trunk disease detection, where the availability of labeled images is particularly limited [47]. Furthermore, data enhancement techniques can be used to enhance datasets; they do not increase the number of classes, which leaves many datasets with low inter-class variability. To address data limitations, transfer learning provides an effective solution. In fact, most of the models mentioned above are fine-tuned with off-the-shelf models, which is one of the most significant advantages of deep learning [98]. Researchers can solve similar problems using models that have already been trained on a large amount of data, and they only need to fine-tune the models using the dataset of the target task to improve the predictive power of the models. In the above study, some researchers applied pre-trained networks, such as SegNet and VGG, to their own collected datasets to complete the training detection.

The detection performance of deep learning models also relies heavily on the quality of the input image, which is usually affected by the image resolution and the accuracy of the vision system hardware. For example, in a study on detecting the number of apple fruits, Smirnov et al. [99] found that the errors in the experimental results mainly came from inaccurate image segmentation and low-resolution cameras. This result suggested that image quality is a key issue affecting the development of deep learning and that high-precision hardware and resolution can avoid introducing noise and distortion to the image, which can provide more image details and help the deep learning model to better understand the target object. Although hardware accuracy and resolution are low in some cases, the use of deep learning algorithms can compensate for hardware deficiencies to some extent. Factors including cost, detection performance, and practical needs need to be balanced with hardware accuracy and resolution of the image to achieve the best results.

## 6. Challenges and Future Trends

For deep learning, the annotation of datasets is often by far the most difficult task, especially for semantic segmentation, where every pixel needs to be annotated, which is an extremely time-consuming task. Some tasks may be difficult to choose the right label due to their complexity or the amount of labor required to label them [100], making it essential to consider unsupervised learning, semi-supervised learning techniques, and even reinforcement learning. These techniques can significantly reduce the reliance on manual annotations by leveraging unlabeled data. For instance, semi-supervised learning methods combine a small amount of labeled data with a large amount of unlabeled data, thereby reducing the need for extensive manual labeling. Unsupervised learning approaches, such as principal component analysis (PCA) [101], K-means clustering, and GAN [102], have been widely used to identify patterns and structures in unlabeled data, which can assist in clustering and dimensionality reduction. These techniques can help address the inadequacy of datasets and the labor-intensive nature of labeling tasks in monitoring apple tree growth and fruit production. By reducing the labeling burden, these methods not only improve the efficiency of dataset creation but also enhance the generalizability of deep learning models for complex agricultural tasks.

Model integration methods may be able to address the phenomenon of unbalanced dataset categories [103]. For example, when collecting datasets, they can be affected by factors such as season, climate, and weather, so the likelihood of getting a particular dataset is small. The research has shown that a combination of integration techniques and random downsampling techniques is expected to solve this problem.

The application of an unmanned aerial vehicle (UAV) in orchard management provides more possibilities for data collection, especially in large-scale monitoring. Due to the dense planting of orchards and the growth of apple trees to a certain height, manual data collection faces challenges, often resulting in low-quality or incomplete images. With the use of a UAV to acquire image data, camera calibration and image processing techniques are crucial for ensuring the reliability of the acquired data. Factors such as altitude, speed, and atmospheric conditions can significantly affect the quality of images captured by the UAV, leading to issues like image blurring or color distortion. By employing advanced image processing techniques and making appropriate technical adjustments, these challenges can be addressed, thus enhancing the accuracy and reliability of aerial images. Furthermore, to improve the precision of orchard monitoring, integrating multiple sensor technologies with UAV systems presents a viable solution. For instance, high-resolution optical cameras provide detailed visual information, hyperspectral imaging captures subtle changes in plant health [104], and LiDAR can generate precise three-dimensional structural maps [105]. By combining these multimodal data with deep learning techniques, a more comprehensive and accurate analysis of orchard growth and health can be achieved.

Exploring new research dimensions, use long- and short-term memory (LSTM) models [106] and recurrent neural network (RNN) models [107], introducing a temporal dimension and a memory function [108]. This allows the use of previously recorded data to predict the growth of apple trees, thus effectively predicting parameters, such as fruit size and yield.

The choice of image format directly affects the performance of recognition and detection tasks. It was found that most of the related literature on apple tree growth and fruit quality mainly focuses on the application of RGB images due to the easy access, low cost, and high processing efficiency of RGB images, as well as the more mature application of existing deep learning algorithms on RGB images. However, multispectral data can provide richer feature information, which helps to improve detection accuracy. Therefore, although currently relying mainly on RGB images, future research will further explore and apply multispectral images to enhance recognition and detection.

The high accuracy of deep learning networks often requires many parameters and operations, which severely hampers development research on mobile devices. So far, most of the studies have mainly focused on the detection accuracy of the models and lacked experimental data related to actual deployment. Future research can further explore lightweight optimization strategies, such as model quantization, pruning [109], knowledge distillation, compressed convolutional kernel filter, and matrix decomposition [110], to minimize the computational burden while ensuring model accuracy. In addition, the use of hardware acceleration schemes such as TensorRT and ONNX Runtime can further enhance the inference speed and improve the usefulness of the model in agricultural intelligent systems. This is particularly important for orchard managers and contributes to the wide application of intelligent systems in agricultural production.

## 7. Conclusions

This study reviews the application of deep learning combined with computer vision for monitoring apple tree growth as well as fruit production in five sections: pests, diseases (located on leaves, fruits, and trunks), organ growth (including fruits, apple blossoms, and branches), yield, and post-harvest detection of fruit defects, and summarizes the current state of the research in each of these sections. Deep learning combined with computer vision techniques performs particularly well in real-time detection tasks compared to previous detection methods. This study discusses the advantages and disadvantages of deep learning, as well as points out some limitations of deep learning techniques applied to apple tree growing as well as real-time detection of fruit production, and proposes methods to address these issues. In conclusion, the combination of deep learning and computer vision shows great potential in real-time monitoring of apple tree growth, which is expected to automate the production of fruit trees and greatly contribute to the prosperity of the orchard industry.

## Figures and Tables

**Table 1:** Summary of deep learning combined with computer vision in apple pest identification.

Pests	Image Type	Number of Images	Model	Accuracy	References
Codling moth and general insect	RGB	4400	Improved LeNet	98.50%	[22]
Codling moth and general insects	RGB	1300	Improved VGG16	94.38%	[24]
Codling moth	RGB	1200	LeNet VGG16	98.30% 88.20%	[23]
Cydia pomonella	RGB	5869 545 500	Double attention-based MobileNetv2	96.61% 99.08% 91.60%	[19]
Leucoptera malifoliella	RGB	4700	EfficientDet	98.00%	[27]
Moth, Pheromone lure, Carpocapsa	RGB	18,300	DNN	94.80%	[26]
Pests	RGB	4440	AlexNet	99.53%	[20]
Nine pests	RGB	6626	PestLite based on YOLOV5	90.70%	[21]
Adhesive pests	RGB	1080	GMM-DC and the improved Mask-RCNN	96.75%	[28]

**Table 2:** Summary of deep learning combined with computer vision for disease detection.

Location of Diseases	Image Type	Name of Diseases	Number of Images	Model	Accuracy	References
Fruit	RGB, HSV	Apple ring rot	5010	DMCNN	99.50%	[34]
	RGB	Anthracnose	640	YOLOV3	IOU = 91.70% at a dataset of 700 images	[31]
	RGB	Marssonia blotch, Alternaria leaf spot, Anthracnose	2945	CNN	99.78%	[32]
	RGB	Rot, Scab, Blotch	319	DCNN	99.99%	[33]
Leaf	RGB	Cedar apple rust, Apple scab, Multiple diseases	3642	An ensemble of pre-trained DenseNet121, EfficientNetB7, and EfficientNet NoisyStudent	96.25%	[42]
	RGB	Rust, Scab, Blotch	6268	YOLOX-ASSANano	MAP = 91.08%	[38]
	RGB	General scab, Serious scab, Grey spot, Rust, Serious cedar rust	2462	Densenet-121 DNN with regression, Densenet-121 DNN with multi-label classification, Densenet-121 DNN with focal loss function	93.51%, 93.31% 93.71%	[37]
	RGB	Marssonia blotch, Alternaria	404	ROI-based DCNN	84.30%	[35]
	RGB	Marsonina coronaria, Scab	20,000	CNN	99.20%	[36]
	RGB	Rust, Scab, Multiple diseases	3651	TFLite	91.00%	[41]
	RGB	Scab, Black rot, Rust	4562	Improved VIT	MAP = 84.00%	[40]
	RGB	Alternaria blotch, Grey spot, Rust	3900	YOLOV5-CBAM-C3TR	MAP = 73.40%	[39]
	RGB	Rust, Scab, Grey spot, Frog eye leaf spot, Powdery mildew, Alternaria blotch	14550	ELM-YOLOV8n	MAP = 96.70%	[45]
	RGB	Rot, Rust, Scab	9395	Improved ResNeXt model	98.94%	[43]
	RGB	Alternaria blotch, Brown spot, Grey spot, Mosaic, Rust	2644	PSPNet-SA	MIOU=97.50% at 1/2 annotated data, MIOU=97.40% at 1/4 annotated data, MIOU=96.50% at 1/8 annotated data	[46]
Trunk	RGB	Round sickness, Rot	3035	VGG19	94.50%	[47]
	RGB	Ring rot, Apple scab	5390	CNNDNN-BiLSTM	88.00%	[48]

**Table 3:** Summary of deep learning detection of apple organs.

Organ	Image Type	Number of Images	Model	Accuracy	References
Flower	RGB	147	CNN	92.70%	[52]
	RGB	205	Mask-RCNN	86.00%	[54]
	RGB	100 18 24 18	DeepLab-ResNet	IOU = 71.40% IOU = 63.00% IOU = 59.00% IOU = 75.40%	[53]
	RGB	37,890	GM-EfficientDet-D5	90.01%	[55]
	RGB	3005	YOLOV5s-ShuffleNetv2-Ghost	91.80%	[56]
	RGB	2200	Improved YOLOV7	80.10%	[57]
Branch	RGB, Point Cloud	300	SegNet	IOU = 67.00%	[58]
	RGB	509	SegNet	94.00%	[59]
	RGB, Depth images	521	U-Net, DeepLabv3, Pix2Pix Generator	IOU = 83.00% IOU = 83.70% IOU = 80.20%	[60]
Fruit	RGB	172	CROP based on U-Net	97.50% at 0.5 IOU	[65]
	RGB	4800	Improved YOLOV3	81.70% at F1 score	[64]
	RGB	2403	CNN	99.89%	[63]
	RGB	20,000	YOLOV5	97.00%	[67]
	RGB	4000	YOLOV5	96.30%	[68]

**Table 4:** Summary of deep learning for predicting apple orchard yield.

Image Type	Number Of Images	Model	Accuracy	References
RGB	958	CNN	95.56% for Dataset1 97.81% for Dataset2 97.83% for Dataset3	[72]
RGB	2268	Tiled Faster R-CNN	90.00%	[77]
RGB	10GB of video	YOLOV7 + MAM	92.00%	[75]
RGB	6700	CNN-SVM	99.70%	[73]
RGB	4246	YoloV7-CA	91.30%	[74]
RGB	2071	AppleYOLO	MAP = 98.50%	[76]

**Table 5:** Summary of deep learning for detecting apple fruit defects.

Image Type	Number of Images	Model	Accuracy	References
RGB	36,000	CNN	99.00%	[82]
RGB	11,020	OB-Net based on a dual-branch structure	95.64%	[84]
RGB	452	YOLOV3	MAP = 74.00%	[93]
RGB	224	SSD	MAP = 87.80%	[81]
RGB	651	U-Net based on CNN, Deeplab based on CNN	mIOU = 99.71%, mIOU = 99.99%	[83]
AC, DC	568	CNN	98.00%	[78]
RGB	500	ASDINet	98.80%	[85]
RGB, NIR	2000	AlexNet, Inception V3, VGG16	RGB:74.66%, 79.33%, 86.00% NIR:99.33%,100.00%, 100.00%	[86]
RGB	5000	Improved model based on deep learning	93.00%	[87]
RGB	2400	SMC-YOLOV8n	mAP = 91.40%	[89]
RGB, AC, DC, RT	8000	YOLOV8n	99.12%	[90]
RGB	800	Improved YOLOV8 model	MAP = 95.30%	[88]

## Data Availability

Not applicable.

## Abbreviations

The following abbreviations are used in this manuscript:

YOLO	You Only Look Once
Faster R-CNN	Faster region-based convolutional network
AlexNet	Alex network
ResNet	Residual network
SegNet	Segmentation network
Mask R-CNN	Mask regional convolutional neural network
RGB	Red-green-blue
3D	Three-dimensional
AI	Artificial intelligence
CNN	Convolutional neural networks
MLP	Multilayer perceptron
DNN	Deep neural networks
SVM	Support vector machines
LRC	Logistic regression classifier
RBF	Radial basis function
RF	Random forest
VGG	Visual geometry group
MTSPPF	Multi-level spatial pyramid pooling
SPPF	Spatial pyramid pooling fusion
ECA	Efficient channel attention
CARAFE	Content-aware reassembly of features
MAP	Mean average precision
ANN	Artificial neural network
PMD	Pest monitoring device
GMM-DC	Density curvature weighted Gaussian mixture model
VIT	Vision transformer
IOU	Intersection over union
KNN	K-nearest neighbors
DCGAN	Deep convolutional generative adversarial network
DCNN	Deep convolutional neural network
DMCNN	Dual-channel convolutional neural network
HSV	Hue-saturation-value
ROI	Region of interest
CBAM	Convolutional block attention module
C3TR	Convolution 3 transformer
EMA	Exponential moving average
SENet	Squeeze-and-excitation networks
RGR	Region growth segmentation
CA	Coordinate attention
CROP	Central roundish object painter
GAN	Generative adversarial network
MS-MLP	Multi-scale multilayered perceptron
WS	Watershed segmentation
CHT	Circular hough transform
PANet	Path aggregation network
MAM	Multi-attention mechanism
IOT	Internet of things
DC	Direct current
AC	Alternating current
SIRI	Structured-illumination reflectance imaging
MIOU	Mean intersection over union
ASDINet	Apple surface defect detection network
GDM	Gradient descent method
SOTA	State-of-the-art
NIR	Near-infrared
PSPNet-SA	Pyramid scene parsing network self-attention
HRNet	High-resolution network
WIOU	Weighted intersection over union
SPD-Conv	Space-to-depth convolution
MSDA	Multi-scale empty attention
CGFPN	Context-guided feature pyramid network
RT	Ratio transformation
PCA	Principal component analysis
UAV	Unmanned aerial vehicle
LSTM	Long- and short-term memory
RNN	Recurrent neural network
